# Regulated Production of CXCL13 within the Central Nervous System

**DOI:** 10.4172/2155-9899.1000460

**Published:** 2016-10-05

**Authors:** David N. Irani

**Affiliations:** Department of Neurology, University of Michigan Medical School, Ann Arbor, Michigan, USA

**Keywords:** CXCL13, Lymphoid chemokines, CXCR5, Ectopic lymphoid follicles, Brain

## Abstract

The chemokine, C-X-C motif ligand 13 (CXCL13), is constitutively expressed in lymphoid organs and controls the recruitment and compartmentalization of lymphocytes and antigen presenting cells within these specialized structures. Recent data, however, also show induction of this molecule under a variety of circumstances during central nervous system (CNS) inflammation. While its role(s) in the pathogenesis of neoplastic, infectious and autoimmune disorders of the CNS remain incompletely understood, growing evidence suggests that CXCL13 could become a relevant therapeutic target in at least some of these conditions. This review focuses on the diseases, cellular sources and external factors known to regulate CXCL13 production in the inflamed CNS.

## CXCL13 Helps Orchestrate the Structure and Function of Secondary Lymphoid Tissues

Lymphoid chemokines are constitutively expressed in lymphoid organs and control the recruitment and compartmentalization of both lymphocytes and antigen presenting cells (APC) within these specialized structures [1–12]. C-C motif ligand (CCL) 19 and CCL21 bind to C-C motif receptor (CCR) 7 and recruit T cells and dendritic cells (DC) to T cell areas of secondary lymphoid tissues [2,3]. C-X-C motif ligand (CXCL) 12 binds to C-X-C motif receptor (CXCR) 4 and attracts multiple immune cell types to lymph nodes and spleen, and along with CXCL13, drives the formation of germinal centers (GC) [11,12]. CXCL13 is made by stromal cells and follicular DC (FDC) in B cell follicles, and recruits B cells and CD4^+^ T follicular helper (Tfh) cells to these compartments via its cognate receptor, CXCR5 [1,3,8,9–12]. Induction of the lymphoid chemokines in both T and B cell areas of lymphoid organs depends on lymphotoxin (LT)-β and tumor necrosis factor (TNF)-α signaling in stromal cells and FDC, both of which are important physiologic sources of these chemoattractant molecules in these tissues [4–6].

Mice genetically deficient in CXCL13 lack most of their lymph nodes and Peyer.s patches from early development [1,8]. Splenic architecture is also disrupted [1], and while circulating B cell numbers are slightly elevated, B cell homing to the peritoneal cavity is reduced in these hosts [1,13]. When normal adult mice are given neutralizing anti-CXCL13 antibodies and immunized with a foreign antigen 24 hours later, splenic follicles rapidly disappear, B cells no longer efficiently surround splenic T cell zones, and FDC become difficult to find [14]. Nonetheless, B cell numbers within GC are preserved, and treated animals mount normal antigen-specific antibody responses [14]. These data show that organized GC in the spleen are not absolutely required for normal humoral immunity, and/or that other lymphoid organs somehow act to maintain systemic antibody responses. Indeed, CXCL13 neutralization, unlike genetic ablation, has no effect on GC size, number or distribution in Peyer’s patches [14].

## CXCL13 Participates in Non-lymphoid Tissue Inflammation

Beyond their role in the development and maintenance of lymphoid tissues, the lymphoid chemokines also drive non-lymphoid tissue inflammation. Ectopic structures resembling the GC of secondary lymphoid organs are found directly in the synovia of patients with rheumatoid arthritis and in the salivary glands of patients with Sjögren’s disease [15]. Experimental evidence supporting a role for the lymphoid chemokines in driving “lymphoid neogenesis” comes in part from studies using transgenic mice where overexpression of CCL21 or CXCL13 in organs such as the pancreas or thyroid gland is sufficient to generate ectopic lymphoid structures and cause diabetes or thyroiditis, respectively [16–19]. CXCL13 is found in naturally occurring gastric mucosa-associated lymphoid structures that develop in response to chronic *Helicobacter pylori* infection [20], and CXCL13-CXCR5 interactions contribute to the development of both *H. pylori*-associated gastritis and gastric lymphomas [20–23]. Thus, ectopic lymphoid follicles that form via local expression of CXCL13 could be a natural feature of chronic organ-specific inflammation [15,24–27]. Under what circumstances CXCL13 gets induced in the CNS, what cells can express this mediator in different disease settings, and how its expression is regulated during local inflammation will be considered here.

## Human Diseases Where CXCL13 is Induced in the CNS

### Primary CNS lymphoma (PCNSL)

PCNSL is a rare but often fatal form of non-Hodgkin B cell lymphoma that arises directly within the CNS. Smith et al. first reported CXCL13 expression by malignant B cells in PCNSL [28], a finding since reproduced by multiple other groups [29–31]. Fischer et al. compared serum and cerebrospinal fluid (CSF) concentrations of both CXCL12 and CXCL13 in a cohort of 30 patients with PCNSL against samples from 40 non-lymphoma controls (10 with and 30 without other CNS malignancies). While neither serum nor CSF CXCL12 levels differed between the CNS lymphoma patients and controls, CSF CXCL13 concentrations were significantly higher in the CNS lymphoma cohort, even as serum CXCL13 levels were consistently low in both groups [32]. Furthermore, when CSF CXCL13 concentrations were followed longitudinally in 7 patients with PCNSL, levels declined in all 5 individuals who responded to chemotherapy and increased in both cases where lymphoma progression occurred [32]. In a separate cohort of patients, Rubenstein et al. also found that mean CSF CXCL13 levels were significantly higher in patients with CNS lymphoma compared to a range of controls, and that progression-free survival with standard treatment was significantly longer for those patients with lower compared to higher CSF levels of this mediator [33]. For the 5 PCNSL patients in this cohort with undetectable CSF CXCL13 levels at diagnosis, none progressed after treatment over a median follow up of 46 months [33]. Thus, multiple studies suggest that CXCL13 contributes to PCNSL pathogenesis and that higher intrathecal levels of this mediator signify more aggressive disease.

### Lyme neuroborreliosis (LNB)

Lyme disease is caused by infection with the tick-borne spirochete, *Borrelia bergdorferi*. An array of CNS complications, collectively referred to as LNB, can include meningitis, encephalopathy, cranial nerve palsies, myelitis and polyradiculitis [34,35]. Neuropathological studies have found structures resembling ectopic GC in the CNS of LNB patients at autopsy [36]. Since then, multiple studies have shown that CXCL13 levels in the CSF of LNB patients can be extremely high [37–43]. Furthermore, when the composition of CSF inflammatory cell infiltrates from LNB patients is analyzed by flow cytometry, CXCL13 levels correlate closely with B cell recruitment to the intrathecal compartment [37,42]. CSF CXCL13 levels typically fall with successful antimicrobial treatment of LNB [38,43]; persistent elevations suggest that the pathogen has evaded clearance and remains infective. Finally, while local B cell immunity supports the clearance of *B. bergdorferi* from the CNS [44–48], self-reactive antibodies can also emerge with notable frequency in the setting of chronic infection [49–51]. Some of these antibodies react to ganglioside epitopes that may be shared between the pathogen and neural tissues [52], but others are specific for myelin or neuronal proteins whose emergence cannot be readily explained by molecular mimicry [50,51]. If B cells making these antimyelin antibodies clonally expand within the CNS as one study suggests [51], then factors controlling their generation, recruitment, and persistence in the brain of LNB patients require further study. It is plausible that CXCL13 contributes to these events.

### Multiple sclerosis (MS)

CXCL13 has been found in local B cell aggregates that develop in the inflamed meninges of a subset of patients with progressive MS [24–26]. These ectopic follicle-like structures contain proliferating B cells, plasma cells, T helper (Th) cells and FDC, their presence in one study correlates directly with adjacent cortical demyelination, neuronal loss and more rapid disease progression [53]. Quantitative PCR analyses of autopsy tissues demonstrate that *cxcl13* mRNA is also induced directly within active demyelinating MS lesions, and immunohistochemistry confirms the presence of CXLC13 protein expression in these lesions [54]. Finally, CSF levels of CXCL13 are increased in patients with the relapsing-remitting form of MS compared to controls, and levels are significantly higher during active disease relapses and decline following the successful application of B cell-directed therapies [54–59]. Even in cases of progressive MS where the long-term clinical benefits of such therapies remain less well established, CSF levels of CXCL13 still consistently fall after 6 months of systemic treatment with a compound that blocks lymphocyte entry into the CNS [60]. These data suggest that changes in the intrathecal concentration of CXCL13 during MS may reflect lymphocyte traffic through the CNS more so than local glial or meningeal production, and that such changes may serve as a biomarker of treatment responsiveness across the spectrum of MS subtypes [61].

## CXCL13 Can be Pathogenic During Neuroinflammation in Experimental Animals

### Experimental autoimmune encephalomyelitis (EAE)

Data generated in EAE, the principal animal model of human MS, demonstrate that CXCL13 plays an actual role in disease pathogenesis rather than simply being a biomarker of ongoing neuroinflammation. Several groups have found CXCL13 in B cell aggregates that develop in the inflamed meninges of mice with EAE [62,63]. Columba-Cabezas et al. showed that treatment with an LTβ receptor (LTβR)-immunoglobulin (Ig) fusion protein that blocks LTβ-LTβR interactions inhibits CXCL13 induction, suppresses the formation of CNS lymphoid follicles and ameliorates disease symptoms in mice with established EAE [64]. Around the same time, Bagaeva et al. showed that EAE onset occurs normally in CXCL13 knockout (KO) mice, but that disease severity wanes over time compared to wild-type (WT) mice because myelin-specific CD4^+^ T cell responses are not sustained [65]. These investigators also showed that systemic administration of a neutralizing anti-CXCL13 antibody to mice at disease onset caused a similar attenuation of symptoms [65]. Rainey-Barger et al. confirmed that CXCL13 deficiency leads to waning numbers of myelin-reactive Th1 and Th17 cells in the periphery of mice after peak EAE, and also showed that CXCL13 KO mice have no defects in the early recruitment of B cells into the inflamed CNS [66]. Since then, a human anti-CXCL13 monoclonal antibody has been developed; it also binds and neutralizes rodent and primate CXCL13 [67]. Not only does this reagent ameliorate EAE induced by CXCR5^+^ Th17 cells *in vivo*, but it also reduces the number of ectopic follicles detected in several different autoimmune disease models, and it interferes with B cell traffic to target areas of the spleen and lymph nodes in adoptive transfer studies [67].

### Other CNS disease models

In a very different experimental system, Jiang et al. showed that CXCL13 induction plays an essential role in driving CXCR5-mediated astrocyte activation and promoting neuropathic pain following experimental spinal nerve ligation [68]. Conversely, CXCL13 plays an indifferent role in the outcome of mice with lethal alphavirus encephalitis [69], and it may even facilitate a beneficial B cell response in the brain following experimental stroke [70]. These data reveal that CXCL13 serves distinct roles in different disease models, and they suggest that targeting the CXCL13-CXCR5 pathway may be most beneficial in the setting of CNS autoimmunity.

## Cellular Sources of CXCL13 in the Inflamed CNS

### Human autopsy tissues

In patients with PCNSL, CXCL13 is made primarily by the malignant B cells that form the tumor itself [28–31]. These cells are also likely the main source of CXCL13 that accumulates in the CSF, and intra-tumoral production of this mediator is believed to support the local recruitment of CXCR5^+^ tumor-infiltrating lymphocytes [28–31]. In some PCNSL specimens, CXCL13 staining is also found in cerebrovascular endothelial cells [28], but whether this demonstrates actual cellular production versus transcellular expression at the blood-brain barrier is more difficult to discern. In MS tissues, immunostaining reveals that both perivascular mononuclear cells and endogenous microglial cells express CXCL13 protein directly within active MS plaques [54], while CXCL13 labeling is most consistent in stromal cells within meningeal B cell aggregates [24–26]. Convincing astrocytic or neuronal production of CXCL13 is not reported in human disease specimens to date, but this may reflect a lack of study in other clinical settings.

### Non-human primates (NHP)

NHP have been used to model human LNB; neurological involvement in rhesus macaques consistently follows intradermal or intracisternal challenge with a neurotropic *B. bergdorferi* isolate [71,72]. In this system, direct inoculation of *B. bergdorferi* into the cisterna magna of rhesus macaques elicits a rapid lymphocytic and monocytic pleocytosis in the CSF, accompanied by rising interleukin (IL)-6, IL-8, CCL2 and CXCL13 levels [73]. When brain tissues from these animals are examined, CXCL13 is found mostly in endogenous microglia and vascular endothelial cells, as well as a few infiltrating macrophages, DC and T cells [73]. Using cultured brain slices prepared from NHP, direct in vitro exposure to *B. bergdorferi* causes microglia to rapidly produce a range of immune mediators, including CXCL13 [74]. As for other experimental systems, EAE can be provoked in common marmosets and in some ways more closely resembles human MS than do its rodent counterparts [75]. Still, while focal areas of cortical demyelination with overlying plasma cells are described in marmoset EAE [76,77], related CXCL13 expression has not been reported. Nonetheless, resident microglia and tissue-infiltrating mononuclear cells appear to be the main parenchymal sources of CXCL13 during neuroinflammatory responses in NHP models when it has been examined.

### Rodents

**Figure 1:**
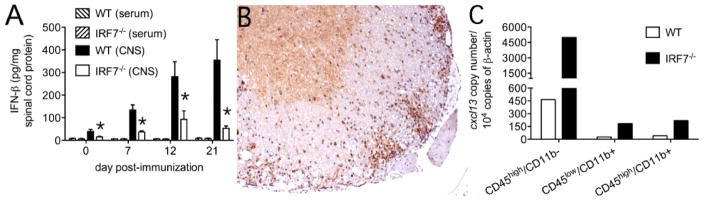
Non-myeloid hematopoietic cells are the main producer of CXCL13 in the spinal cords of mice at peak EAE, and local production of this mediator is greatly augmented in animals lacking IRF7. (A) IRF7 KO mice mount a deficient type-I IFN response within the CNS over the course of EAE compared to WT controls. ^*^p<0.05 comparing levels in WT *vs*. IRF7 KO mice at each time point (n=5 mice per group). (B) Histochemical staining for CXCL13 at peak EAE shows prominent protein expression (brown) directly within white matter inflammatory infiltrates of an IRF7 KO mouse. (C) When immune cells are pooled from the spinal cords of WT or IRF7 KO mice at peak disease, sorted by flow cytometry into distinct subsets based on patterns of CD45 and CD11b expression as described [69], and extracted RNA used for quantitative PCR expression of *cxcl13* copy number, transcript levels are highest in CD45^high^/CD11b-cells (non-myeloid hematopoietic cells) compared to either CD45^high^/CD11b+ cells (infiltrating myeloid cells) or CD45^low^/CD11b+ cells (endogenous microglia). *cxcl13* transcripts are much more abundant in each IRF7 KO compared to WT cell type.

In a spinal nerve ligation model of neuropathic pain, Jiang et al. showed that CXCL13 gets persistently induced in spinal cord neurons and leads to the generation of pain behaviors [68]. During experimental viral encephalomyelitis where neurons are the main disease target, *CXCL13* is instead made by microglia [69]. In the meninges of mice with EAE bearing ectopic lymphoid follicles, CXCL13 is reported along collagen networks and within infiltrating FDC [62,63]. In spinal cord parenchyma, we find that tissue-infiltrating CD45^+^/CD11b^−^ cells are the main source of cxcl13 mRNA, being some 10-fold higher in the setting of an impaired type-I IFN response (Figure 1). Although the identity of these non-myeloid hematopoietic cells making CXCL13 remains to be elucidated, histochemical staining confirms robust CXCL13 expression directly within white matter inflammatory infiltrates (Figure 1). Ongoing studies aim to determine whether these cells represent some innate lymphoid cell that may predispose to ectopic lymphoid follicle formation.

## Signaling Pathways and Mediators Known to Regulate CXCL13 Expression in the CNS

### Inducers

**Figure 2:**
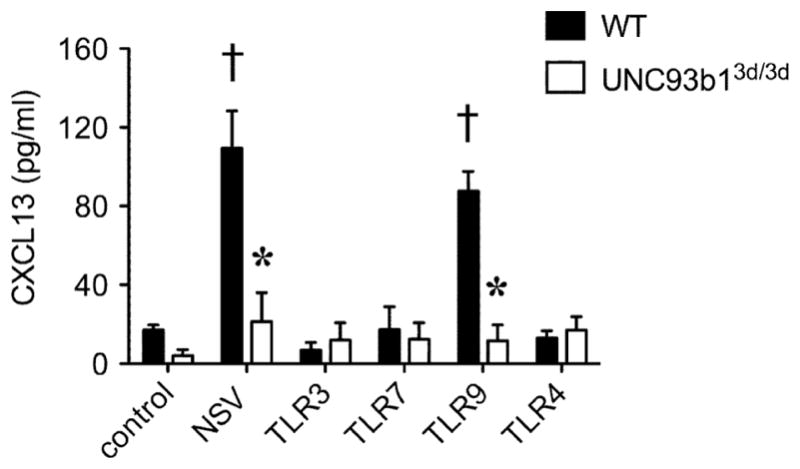
Primary microglia produce CXCL13 in response to certain stimuli *in vitro*, and production depends on the endoplasmic reticulum protein, UNC93b1. Cultures were prepared and assays were performed as described [80]. Both neuroadapted Sindbis virus (NSV) and a synthetic TLR9 ligand, but not ligands for the other TLRs examined, stimulate CXCL13 production compared untreated control cells. ^†^p<0.05 comparing levels in treated *vs*. untreated control WT cultures (n=5 replicates per group). When cells prepared from mice bearing a “triple D” (3d) mutation of UNC93b1 [81], a protein that delivers nucleotide-sensing TLRs to endolysosomes [82], are tested, this stimulated CXCL13 production is completely ablated. ^*^p<0.05 comparing WT *vs*. mutant UNC39b1 (UNC93b1^3d/3d^) responses under each stimulation condition (n=5 replicates per group).

Both LTβ and TNFα signaling induce CXCL13 expression by stromal cells in the spleen [4–6]. Similarly, LTβ signaling appears to be important for CXCL13 production in the CNS during EAE, since an LTβR-Ig fusion protein inhibits expression of this mediator and suppresses formation of ectopic lymphoid follicles in the meninges of diseased animals [64]. We find that stimulating primary murine microglia via particular Toll-like receptors (TLR) but not others can trigger CXCL13 release in vitro (Figure 2). Furthermore, a specific endoplasmic reticulum protein (UNC93b1) must successfully convey these activating TLR to endosomes in order for CXCL13 production to occur (Figure 2). Another group reported that Th17 cells, but not Th1 or Th2 cells, can express CXCL13 both *in vitro* and *ex vivo* [78], although the signaling pathway(s) and transcription factor(s) supporting production of this mediator in these cells remain undefined. Th17 cell differentiation is remarkably plastic, and it is plausible these cells morph into something that more closely resembles an innate lymphoid cell before any CXCL13 production can begin.

### Suppressors

In the lone experimental setting where neuronal expression of CXCL13 is reported, a particular microRNA (miR-186-5p) has been confirmed as a CXCL13 suppressor [68]. Spinal nerve ligation causes a loss of miR-186-5p expression that, in turn, allows CXCL13 induction in injured neurons and leads to the development of neuropathic pain [68]. In a traumatic brain injury (TBI) model, Huang et al. recently found that the NG2 proteoglycan, perhaps best known as a marker of glial progenitor cells, suppresses CXCL13 expression by microglia around the lesion edge and renders these cells less likely to express the canonical M2 marker, Arginase 1 [79]. Although NG2 KO mice have a worse outcome from TBI compared to WT controls, an underlying role for CXCL13 in this setting remains unproven [79].

**Figure 3:**
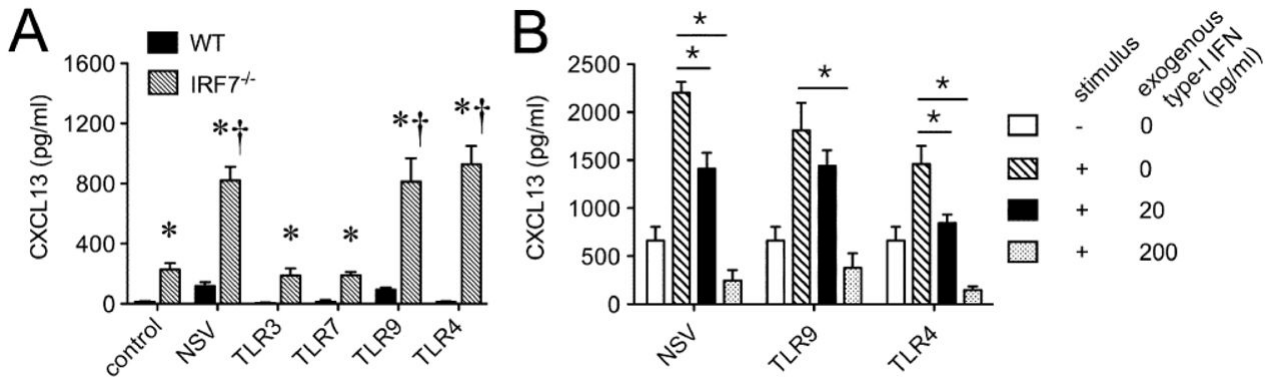
Primary microglia from IRF7 KO mice produce more CXCL13 that WT cells, and production by KO cells is suppressed by exogenous type-I IFN in a dose dependent manner. Cultures were prepared and assays were performed as described [69]. (A) Neuroadapted Sindbis virus (NSV) as well as synthetic TLR9 and TLR4 ligands trigger enhanced CXCL13 production compared to untreated cells. ^†^p<0.05 comparing levels in treated *vs*. untreated control IRF7 KO cultures (n=5 replicates per group). When WT and IRF7 KO cells are compared to one another, CXCL13 production is markedly enhanced in the KO cells. ^*^p<0.05 comparing WT *vs*. IRF7 KO responses under each stimulation condition (n=5 replicates per group). (B) When IRF7 KO microglia are treated with either NSV, a TLR9 ligand or a TLR4 ligand, exogenous IFNβ suppresses stimulated CXCL13 production in a dose dependent manner. ^*^p<0.05 comparing stimulated responses in cultures without *vs*. with exogenous IFNβ.

Our data in show that cxcl13 transcription is markedly enhanced in both CNS-resident and CNS-infiltrating immune cells derived from interferon response factor (IRF) 7 KO mice that also fail to generate a normal endogenous type-I IFN response during EAE (Figure 1). When primary microglia are prepared from both WT and IRF7 KO mice and stimulated *in vitro*, not only is CXCL13 production enhanced in the KO cells by stimuli that also provoke some CXCL13 production by WT cells, but it is also made by KO cells following exposure to a stimulus that does not trigger its release by WT cells (Figure 3). Furthermore, this CXCL13 over-production by IRF7 KO microglia can be suppressed by exogenous type-I IFN (Figure 3), and is reproduced by primary cells derived from type-I IFN receptor KO mice [69]. These data show that type-I IFNs are suppressors of CXCL13 production across a range of immune cell types.

## Conclusions

CXCL13 gets produced within the CNS during a range of human diseases, and it directly contributes to disease pathogenesis in several related animal models. Most current data point to local CNS production of CXCL13 by endogenous microglia and tissue-infiltrating myeloid cells, although emerging evidence suggests that both stromal cells in the meninges and infiltrating lymphoid cells can also be sources of this mediator under certain circumstances. Expected innate immune stimuli (LTβ, certain infectious pathogens) can provoke CXCL13 production within the CNS, and a handful of studies now suggest that both intrinsic (miR-186-5p, NG2) and inducible (type-I IFN) mechanisms can inhibit this event.

## References

[1] Forster R, Matis AE, Kremmer E, Wolf E, Brem G (1996). A putative chemokine receptor, BLR, directs B cell migration to defined lymphoid organs and specific anatomic compartments of the spleen. Cell.

[2] Yoshie O, Imai T, Nomiyama H (1997). Novel lymphocyte-specific CC chemokines and their receptors. J Leukoc Biol.

[3] Legler DF, Loetscher M, Roos RS, Clark-Lewis I, Baggiolini M (1998). B-cell attracting chemokine, a human CXC chemokine expressed in lymphoid tissues, selectively attracts B lymphocytes via BLR1/CXCR5. J Exp Med.

[4] Mackay F, Browning JL (1998). Turning off follicular dendritic cells. Nature.

[5] Fu YX, Chaplin DD (1999). Development and maturation of secondary lymphoid tissues. Annu Rev Immunol.

[6] Ngo VN, Korner H, Gunn MD, Schmidt KN, Riminton DS (1999). Lymphotoxin-α/β and tumor necrosis factor are required for stromal cell expression of homing chemokines in B and T cell areas of the spleen. J Exp Med.

[7] Zlotnik A, Morales J, Hedrick JA (1999). Recent advances in chemokines and chemokine receptors. Crit Rev Immunol.

[8] Ansel KM, Ngo VN, Hyman PL, Luther SA, Forster JD (2000). A chemokine-driven positive feedback loop organizes lymphoid follicles. Nature.

[9] Moser B, Schaerli P, Loetscher P (2002). CXCR5(+) T cells: follicular homing takes center stage in T-helper-cell responses. Trends Immunol.

[10] Müller G, Höpken UE, Lipp M (2003). The impact of CCR7 and CXCR5 on lymphoid organ development and systemic immunity. Immunol Rev.

[11] Campbell DJ, Kim CH, Butcher EC (2003). Chemokines in the systemic organization of immunity. Immunol Rev.

[12] Allen CD, Ansel KM, Low C, Lesley R, Tamamura H (2004). Germinal center dark and light zone organization is mediated by CXCR4 and CXCR5. Nat Immunol.

[13] Ansel KM, Harris RB, Cyster JG (2002). CXCL13 is required for B1 cell homing, natural antibody production, and body cavity immunity. Immunity.

[14] Finch DK, Ettinger R, Karnell JL, Herbst R, Sleeman MA (2013). Effects of CXCL13 inhibition on lymphoid follicles in models of autoimmune disease. Eur J Clin Invest.

[15] Hjelmström P (2001). Lymphoid neogenesis: de novo formation of lymphoid tissue in chronic inflammation through expression of homing chemokines. J Leukoc Biol.

[16] Fan L, Reilly CR, Luo Y, Dorf ME, Lo D (2000). Cutting edge: ectopic expression of the chemokine TCA4/SLC is sufficient to trigger lymphoid neogenesis. J Immunol.

[17] Luther SA, Lopez T, Bai W, Hanahan D, Cyster JG (2000). BLC expression in pancreatic islets causes B cell recruitment and lymphotoxin-dependent lymphoid neogenesis. Immunity.

[18] Luther SA, Bidgol A, Hargreaves DC, Schmidt A, Xu Y (2002). Differing activities of homeostatic chemokines CCL19, CCL2, and CXCL12 in lymphocyte and dendritic cell recruitment and lymphoid neogenesis. J Immunol.

[19] Chen SC, Vassileva G, Kinsley D, Holzmann S, Manfra D (2002). Ectopic expression of the murine chemokines CCL21a and CCL21b induces the formation of lymph node-like structures in pancreas, but not skin, of transgenic mice. J Immunol.

[20] Mazzucchelli L, Blaser A, Kappeler A, Scharli P, Laissue JA (1999). BCA-1 is highly expressed in Helicobacter pylori-induced mucosa-associated lymphoid tissue and gastric lymphoma. J Clin Invest.

[21] Winter S, Loddenkemper C, Aebischer A, Rabel K, Hoffmann K (2010). The chemokine receptor CXCR5 is pivotal for ectopic mucosa-associated lymphoid tissue neogenesis in chronic Helicobacter pylori-induced inflammation. J Mol Med (Berl).

[22] Nakashima Y, Isomoto H, Matsushima K, Yoshida A, Nakayama T (2011). Enhanced expression of CXCL13 in human Helicobacter pyloriassociated gastritis. Dig Dis Sci.

[23] Yamamoto K, Nishiumi S, Yang L, Klimatcheva E, Pandina T (2014). Anti-CXCL13 antibody can inhibit the formation of gastric lymphoid follicles induced by Helicobacter infection. Mucosal Immunol.

[24] Serafini B, Rosicarelli B, Magiozzi R, Aloisi F (2004). Detection of ectopic B-cell follicles with germinal centers in meninges of patients with secondary progressive multiple sclerosis. Brain Pathol.

[25] Magliozzi R, Howell O, Vora A, Serafini B, Nicholas R (2007). Meningeal B-cell follicles in secondary progressive multiple sclerosis associate with early onset of disease and severe cortical pathology. Brain.

[26] Aloisi F, Columba-Cabezas S, Franciotta D, Rosicarelli B, Magliozzi R (2008). Lymphoid chemokines in chronic neuroinflammation. J Neuroimmunol.

[27] Lalor SJ, Segal BM (2010). Lymphoid chemokines in the CNS. J Neuroimmunol.

[28] Smith JR, Braziel RM, Paoletti S, Lipp M, Uguccioni M (2003). Expression of B cell-attracting chemokine 1 (CXCL13) by malignant lymphocytes and vascular endothelium in primary central nervous system lymphoma. Blood.

[29] Brunn A, Montesinos-Rongen M, Strack A, Reifenberger G, Mawrin C (2007). Expression pattern and cellular sources of chemokines in primary central nervous system lymphoma. Acta Neuropathol.

[30] Tun HW, Personett D, Baskerville KA, Menke DM, Jaeckle KA (2008). Pathway analysis of primary central nervous system lymphoma. Blood.

[31] Sugita Y, Terasaki M, Nakashima S, Ohshima K, Morioka M, Abe H (2015). The perivascular microenvironment in primary central nervous system lymphomas: the role of chemokines and endothelin B receptor. Brain Tumor Pathol.

[32] Fischer L, Korfel A, Pfeiffer S, Kiewe P, Volk HD (2009). CXCL13 and CXCL12 in central nervous system lymphoma patients. Clin Cancer Res.

[33] Rubenstein JL, Wong VS, Kadoch C, Gao HX, Barajas R (2013). CXCL13 plus interleukin 10 is highly specific for the diagnosis of CNS lymphoma. Blood.

[34] Pachner AR, Steele AC (1985). The triad of neurologic manifestations of Lyme disease: meningitis, cranial neuritis, and radiculoneuritis. Neurology.

[35] Pachner AR (1988). Borrelia burgdorferi in the nervous system: the new “great imitator”. Ann N Y Acad Sci.

[36] Narayan K, Dail D, Li L, Cadavid D, Amrute S (2005). The nervous system as ectopic germinal center: CXCL13 and IgG in lyme neuroborreliosis. Ann Neurol.

[37] Rupprecht TA, Plate A, Adam M, Wick M, Kastenbauer S (2009). The chemokine CXCL13 is a key regulator of B cell recruitment to the cerebrospinal fluid in acute Lyme neuroborreliosis. J Neuroinflammation.

[38] Senel M, Rupprecht TA, Tumani H, Pfister HW, Ludolph AC (2010). The chemokine CXCL13 in acute neuroborreliosis. J Neurol Neurosurg Psychiatry.

[39] Tjernberg I, Henningsson AJ, Eliasson I, Forsberg P, Ernerudh J (2011). Diagnostic performance of cerebrospinal fluid chemokine CXCL13 and antibodies to the C6-peptide in Lyme neuroborreliosis. J Infect.

[40] Schmidt C, Plate A, Angele B, Pfister HW, Wick M (2011). A prospective study on the role of CXCL13 in Lyme neuroborreliosis. Neurology.

[41] van Burgel ND, Bakels F, Kroes AC, van Dam AP (2011). Discriminating Lyme neuroborreliosis from other neuroinflammatory diseases by levels of CXCL13 in cerebrospinal fluid. J Clin Microbiol.

[42] Kowarik MC, Cepok S, Sellner J, Grummel V, Weber MS (2012). CXCL13 is the major determinant for B cell recruitment to the CSF during neuroinflammation. J Neuroinflammation.

[43] Hytonen J, Kortela E, Waris M, Puustinen J, Salo J (2014). CXCL13 and neopterin concentrations in cerebrospinal fluid of patients with Lyme neuroborreliosis and other diseases that cause neuroinflammation. J Neuroinflammation.

[44] Schoenfeld R, Araneo B, Ma Y, Yang LM, Weis JJ (1992). Demonstration of a B-lymphocyte mitogen produced by the Lyme disease pathogen, Borrelia bergdorferi. Infect Immun.

[45] Cadavid D, O’Neill T, Schaefer H, Pachner AR (2000). Localization of Borrelia burgdorferi in the nervous system and other organs in a nonhuman primate model of lyme disease. Lab Invest.

[46] Razavi-Encha F, Fleury-Feith J, Gherardi R, Bernaudin JF (1987). Cytologic features of cerebrospinal fluid in Lyme disease. Acta Cytol.

[47] Cepok S, Zhou D, Vogel F, Rosche B, Grummel V (2003). The immune response at onset and during recovery from Borrelia burgdorferi meningoradiculitis. Arch Neurol.

[48] Widhe M, Jarefors S, Ekerfelt C, Vrethem M, Bergstrom S (2004). Borrelia-specific interferon-gamma and interleukin-4 secretion in cerebrospinal fluid and blood during Lyme borreliosis in humans: association with clinical outcome. J Infect Dis.

[49] García Moncó JC, Wheeler CM, Benach JL, Furie RA, Lukehart SA (1993). Reactivity of neuroborreliosis patients (Lyme disease) to cardiolipin and gangliosides. J Neurol Sci.

[50] Kaiser R (1995). Intrathecal immune response in patients with neuroborreliosis: specificity of antibodies for neuronal proteins. J Neurol.

[51] Kuenzle S, von Budingen HC, Meier M, Harrer MD, Urich E (2007). Pathogen specificity and autoimmunity are distinct features of antigen-driven immune responses in neuroborreliosis. Infect Immun.

[52] Berndtson K (2013). Review of evidence for immune evasion and persistent infection in Lyme disease. Int J Gen Med.

[53] Haugen M, Frederiksen JL, Degn M (2014). B cell follicle-like structures in multiple sclerosis-with focus on the role of B cell activating factor. J Neuroimmunol.

[54] Krumbholz M, Theil D, Cepok S, Hemmer B, Kivisäkk P (2006). Chemokines in multiple sclerosis: CXCL12 and CXCL13 up-regulation is differentially linked to CNS immune cell recruitment. Brain.

[55] Sellebjerg F, Börnsen L, Khademi M, Krakauer M, Olsson T (2009). Increased cerebrospinal fluid concentrations of the chemokine CXCL13 in active MS. Neurology.

[56] Piccio L, Naismith RT, Trinkaus K, Klein RS, Parks BJ (2010). Changes in B- and T-lymphocyte and chemokine levels with rituximab treatment in multiple sclerosis. Arch Neurol.

[57] Brettschneider J, Czerwoniak A, Senel M, Fang L, Kassubek J (2010). The chemokine CXCL13 is a prognostic marker in clinically isolated syndrome (CIS). PLoS One.

[58] Khademi M, Kockum I, Andersson ML, Iacobaeus E, Brundin L (2011). Cerebrospinal fluid CXCL13 in multiple sclerosis: a suggestive prognostic marker for the disease course. Mult Scler.

[59] Ragheb S, Li Y, Simon K, VanHaerents S, Galimberti D (2011). Multiple sclerosis: BAFF and CXCL13 in cerebrospinal fluid. Mult Scler.

[60] Romme Christensen J, Ratzer R, Börnsen L, Lyksborg M, Garde E (2014). Natalizumab in progressive MS: results of an open-label, phase 2A, proof-of-concept trial. Neurology.

[61] Harris VK, Sadiq SA (2014). Biomarkers of therapeutic response in multiple sclerosis: current status. Mol Diagn Ther.

[62] Magliozzi R, Columba-Cabezas S, Serafini B, Aloisi F (2004). Intracerebral expression of CXCL13 and BAFF is accompanied by formation of lymphoid follicle-like structures in the meninges of mice with relapsing experimental autoimmune encephalomyelitis. J Neuroimmunol.

[63] Peters A, Pitcher LA, Sullivan JM, Mitsdoerffer M, Acton SE (2011). Th17 cells induce ectopic lymphoid follicles in central nervous system inflammation. Nat Immunol.

[64] Columba-Cabezas S, Griguoli M, Rosicarelli B, Magliozzi R, Ria F (2006). Suppression of extablished experimental autoimmune encephalomyelitis and formation of meningeal lymphoid follicles by lymphotoxin? receptor-Ig fusion protein. J Neuroimmunol.

[65] Bagaeva LV, Rao P, Powers JM, Segal BM (2006). CXC chemokine ligand 13 plays a role in experimental autoimmune encephalomyelitis. J Immunol.

[66] Rainey-Barger EK, Rumble JM, Lalor SJ, Esen N, Segal BM (2011). The lymphoid chemokine, CXCL13, is dispensable for the initial recruitment of B cells to the acutely inflamed central nervous system. Brain Behav Immun.

[67] Klimatcheva E, Pandina T, Reilly C, Torno S, Bussler H (2015). CXCL13 antibody for the treatment of autoimmune disorders. BMC Immunol.

[68] Jiang BC, Cao DL, Zhang X, Zhang ZJ, He LN (2016). CXCL13 drives spinal astrocyte activation and neuropathic pain via CXCR5. J Clin Invest.

[69] Esen N, Rainey-Barger EK, Huber AK, Blakely PK, Irani DN (2014). Type-I interferons suppress microglial production of the lymphoid chemokine, CXCL13. Glia.

[70] Monson NL, Ortega SB, Ireland SJ, Meeuwissen AJ, Chen D (2014). Repetitive hypoxic preconditioning induces an immunosuppressed B cell phenotype during endogenous protection from stroke. J Neuroinflammation.

[71] Pachner AR, Delaney E, O’Neill T, Major E (1995). Inoculation of nonhuman primates with the N40 strain of Borrelia bergdorferi leads to a model of Lyme neuroborreliosis faithful to the human disease. Neurology.

[72] Pachner AR, Gelderblom H, Cadavid D (2001). The rhesus model of Lyme neuroborreliosis. Immunol Rev.

[73] Ramesh G, Borda JT, Gill A, Ribka EP, Morici LA (2009). Possible role of glial cells in the onset and progression of Lyme neuroborreliosis. J Neuroinflammation.

[74] Ramesh G, Borda JT, Kaushal D, Ramamoorthy R, Lackner AA (2008). Interaction of the Lyme disease spirochete Borrelia bergdorferi with brain parenchyma elicits inflammatory mediators from glial cells as well as glial and neuronal apoptosis. Am J Pathol.

[75] ‘t Hart BA, van Kooyk Y, Geurts JJ, Gran B (2015). The primate autoimmune encephalomyelitis model: a bridge between mouse and man. Ann Clin Transl Neurol.

[76] Pomeroy IM, Matthews PM, Frank JA, Jordan EK, Esiri MM (2005). Demyelinated cortical lesions in marmoset autoimmune encephalomyelitis mimic those in multiple sclerosis. Brain.

[77] Kramann N, Neid K, Menken L, Schlumbohm C, Stadelmann C (2015). Increased meningeal T and plasma cell infiltration is associated with early subpial cortical demyelination in common marmosets with experimental autoimmune encephalomyelitis. Brain Pathol.

[78] Takagi R, Higashi T, Hashimoto K, Nakano K, Mizuno Y (2008). B cell chemoattractant CXCL13 is preferentially expressed by human Th17 cell clones. J Immunol.

[79] Huang C, Sakry D, Menzel L, Dangel L, Sebastiani A (2016). Lack of NG2 exacerbates neurological outcome and modulates glial responses after traumatic brain injury. Glia.

[80] Esen N, Blakely PK, Rainey-Barger EK, Irani DN (2012). Complexity of the microglial activation pathways that drive innate host responses during lethal alphavirus encephalitis in mice. ASN Neuro.

[81] Tabeta K, Hoebe K, Janssen EM, Du X, Georgel P (2006). The Unc93b1 mutation 3d disrupts exogenous antigen presentation and signaling via Toll-like receptors 3, 7 and 9. Nat Immunol.

[82] Kim YM, Brinkmann MM, Paquet ME, Ploegh HL (2008). UNC93b1 delivers nucleotide-sensing toll-like receptors to endolysosomes. Nature.

